# Cutis laxa and excessive bone growth due to de novo mutations in *PTDSS1*

**DOI:** 10.1002/ajmg.a.38604

**Published:** 2018-01-17

**Authors:** Juliette Piard, James Lespinasse, Marketa Vlckova, Martin A. Mensah, Sorin Iurian, Martina Simandlova, Marcela Malikova, Oliver Bartsch, Massimiliano Rossi, Marion Lenoir, Frédérique Nugues, Stefan Mundlos, Uwe Kornak, Philip Stanier, Sérgio B. Sousa, Lionel Van Maldergem

**Affiliations:** ^1^ Centre de Génétique Humaine Université de Franche‐Comté Besançon France; ^2^ Service de Cytogénétique Centre Hospitalier de Chambéry‐Hôtel Dieu Chambéry France; ^3^ Department of Biology and Medical Genetics Motol Hospital Charles University Prague Czech Republic; ^4^ Institut für Medizinische Genetik und Humangenetik Charité − Universitätsmedizin Berlin corporate member of Freie Universität Berlin Humboldt‐Universität zu Berlin, and Berlin Institute of Health Berlin Germany; ^5^ Faculty of Medicine Lucian Blaga University Sibiu Sibiu Romania; ^6^ Institute of Human Genetics Medical Center of the Johannes Gutenberg University Mainz Mainz Germany; ^7^ Service de Génétique, Hospices Civils de Lyon Centre de Recherche en Neurosciences de Lyon Bron France; ^8^ Service de Radiologie Pédiatrique et Imagerie de la Femme Centre Hospitalier Régional Universitaire de Besançon Besançon France; ^9^ Service d'Imagerie Pédiatrique Centre Hospitalier Universitaire Grenoble Alpes Grenoble France; ^10^ Genetics and Genomic Medicine UCL GOS Institute of Child Health London UK; ^11^ Serviço de Genética Medica Hospital Pediatrico Centro Hospitalar e Universitário de Coimbra Coimbra Portugal

**Keywords:** cutis laxa, hyperostotic skeletal dysplasia, Lenz–Majewski syndrome, *PTDSS1*

## Abstract

The cutis laxa syndromes are multisystem disorders that share loose redundant inelastic and wrinkled skin as a common hallmark clinical feature. The underlying molecular defects are heterogeneous and 13 different genes have been involved until now, all of them being implicated in elastic fiber assembly. We provide here molecular and clinical characterization of three unrelated patients with a very rare phenotype associating cutis laxa, facial dysmorphism, severe growth retardation, hyperostotic skeletal dysplasia, and intellectual disability. This disorder called Lenz–Majewski syndrome (LMS) is associated with gain of function mutations in *PTDSS1*, encoding an enzyme involved in phospholipid biosynthesis. This report illustrates that LMS is an unequivocal cutis laxa syndrome and expands the clinical and molecular spectrum of this group of disorders. In the neonatal period, brachydactyly and facial dysmorphism are two early distinctive signs, later followed by intellectual disability and hyperostotic skeletal dysplasia with severe dwarfism allowing differentiation of this condition from other cutis laxa phenotypes. Further studies are needed to understand the link between *PTDSS1* and extra cellular matrix assembly.

## 1 INTRODUCTION

Congenital cutis laxa (CL) is a rare and heterogeneous condition characterized by over‐folded skin giving a prematurely aged appearance from birth onwards. Based on its accompanying features, it usually falls within a clinical spectrum that to date includes thirteen delineated conditions corresponding to *FBLN5‐, FBLN4‐, ELN‐, ATP6V0A2‐, PYCR1‐, ALDH18A1‐, RIN2‐*, *LTBP4*‐, *ATP6V1E1*‐, and *ATP6V1A*‐related cutis laxa, gerodermia osteodysplastica, occipital horn syndrome, and arterial tortuosity syndrome (OMIM 219100, 614437, 130160, 219200, 614438, 219150, 610222, 613177, 108746, 607027, 231070, 304150, 208050) (Callewaert & Urban, 2016; Loeys, De Paepe, & Urban, 2011; Vanakker, Callewaert, Malfait, & Coucke, 2015; Van Damme et al., 2017; Van Maldergem, Dobyns, & Kornak, 2009; Van Maldergem & Loeys, 2009). The underlying molecular defects in CL perturb extracellular matrix assembly and/or homeostasis and involve all steps in elastic fiber formation. An activation of TGFβ signaling, which plays a crucial role in soft connective tissue and skeletal development, has been observed in several types of CL (MacFarlane, Haupt, Dietz, & Shore, 2017; Renard et al., 2010).

We describe here three patients with Lenz–Majewski syndrome (LMS; OMIM 612792), which is a very rare hyperostotic skeletal dysplasia with intellectual disability (Lenz & Majewski, 1974), belonging to the bona fide congenital CL group of syndromes. LMS is caused by activating de novo heterozygous mutations in *PTDSS1*, encoding phosphatidylserine synthase 1 (PSS1). It is not understood at present why deregulated PSS1 activity in the endoplasmic reticulum can result in skeletal dysplasia and CL (Sousa et al., 2014). The skin abnormality is particularly striking in the three patients described here. One patient was initially referred to the Cutis Laxa Study Group for advice and has been briefly reported in the seminal paper describing the molecular basis of LMS (Sousa et al., 2014). The other two patients described for the first time here, are the eighth and ninth LMS patients reported with a defined *PTDSS1* mutation.

## 2 SUBJECTS

### 2.1 Informed consent

Written informed consent was obtained from the guardians of all patients.

### 2.2 Patient 1

**Figure 1:**
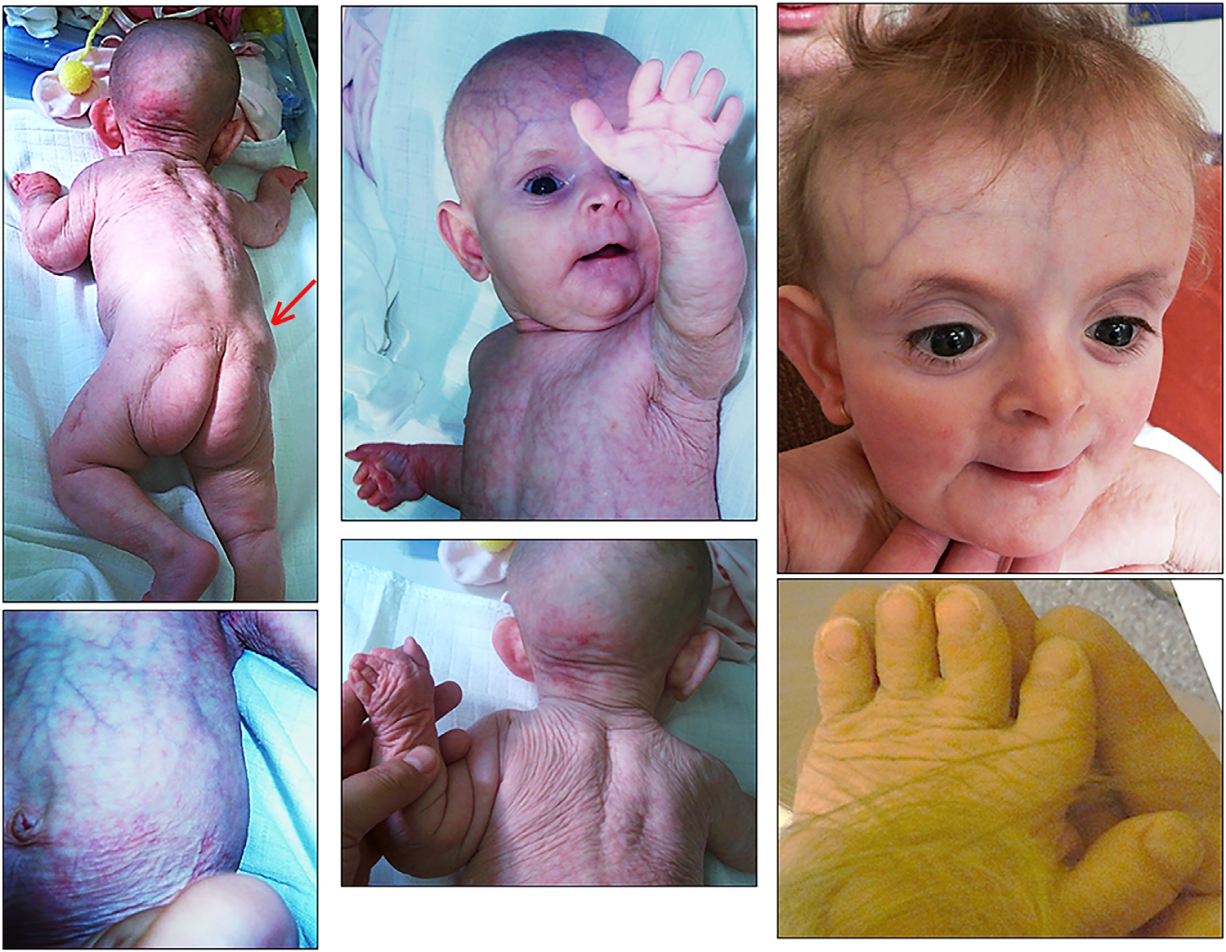
Photographs of patient 1 at 6 months (left panel, middle panel, hand) and 18 months (upper right photograph) showing cutis laxa, prominent veins, lipodystrophy (arrow), brachydactyly and facial dysmorphism. [Color figure can be viewed at http://wileyonlinelibrary.com]

This girl was born at term after an uneventful pregnancy to parents aged 27 and 30 years, originating from Bohemia in the Czech Republic. Her birth weight was 2700 g (−1.8 SD), length was 44 cm (−3.5 SD), and OFC was 32 cm (−3SD). Over‐folded skin affecting the whole body, prominent veins, joint laxity, brachydactyly, and lipodystrophy of buttocks were present. A large fontanelle and a distinct facial dysmorphism including broad forehead, telecanthus, wide mouth, long philtrum, and thin vermilion of the lips were also observed (Figure 1). Her growth and development were severely delayed. At 9 years, she could not walk without assistance, her height was 84 cm (−9 SD), weight was 10.1 kg (−8.7 SD), and OFC was 45.5 cm (−5 DS). She has no speech. Her skin appeared thin, with wrinkles distributed regularly over her whole body. Tooth eruption was delayed. Changes in her facial gestalt were obvious: prognathism, prominent eyes, coarseness, and a progeroid appearance. Sparse hair, oligodontia, and kyphoscoliosis were also noted. Over the years, fading of her CL was seen. Brain MRI showed diffuse cortical atrophy, enlarged lateral ventricles, and apparent pituitary hypoplasia. Bone X‐rays showed structural changes to all bones, with sclerosis mainly in the skull and vertebra, hip dislocation, turricephaly, mildly delayed ossification and thickness of the long bone diaphyses.

### 2.3 Patient 2

**Figure 2:**
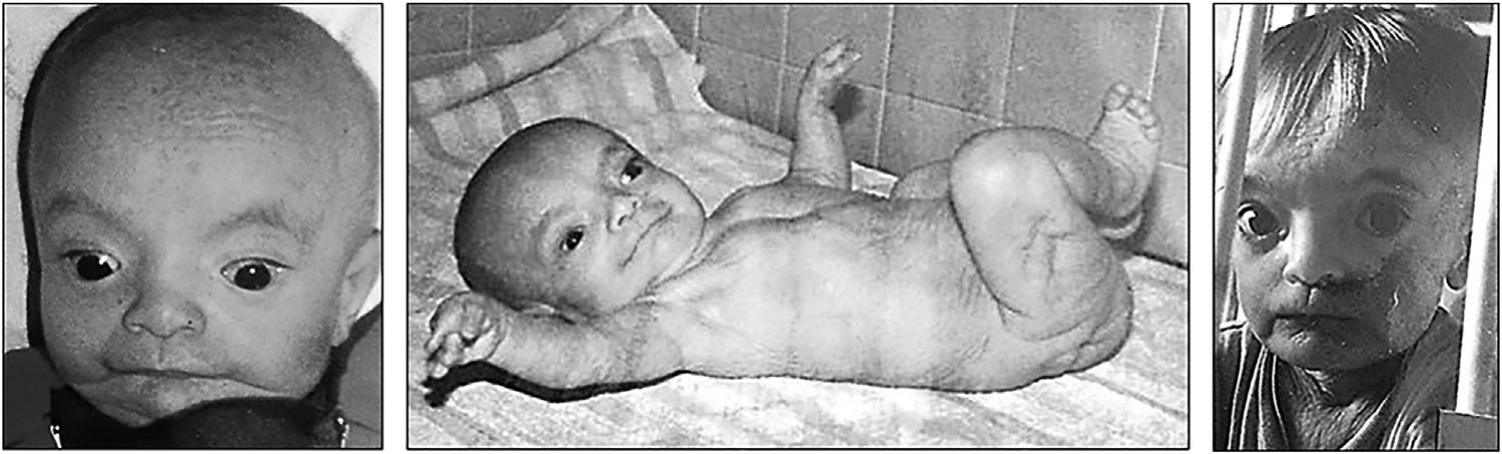
Patient 2: Cutis laxa (notably in the neck), lipodystrophy, prominent eyes, and thin vermilion of the lips are seen in the first year of life

**Figure 3:**
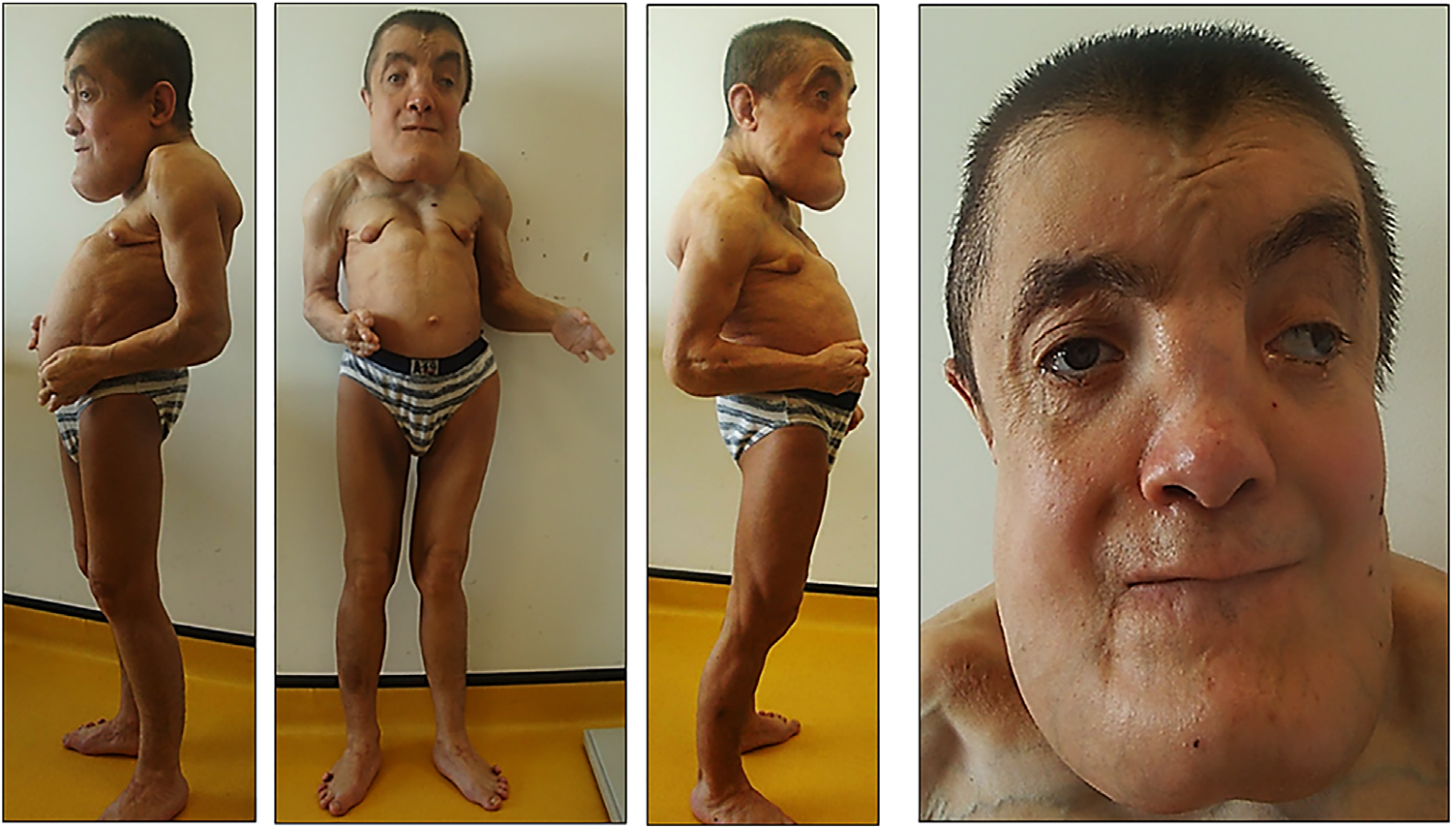
Patient 2: At 25 years, note the tall and broad chin with marked prognathism, long face, broad forehead, high arched thick eyebrows, high and broad nasal root, thin vermilion of the lips, limited extension of the elbow joints, strabismus, and prominent veins. [Color figure can be viewed at http://wileyonlinelibrary.com]

**Figure 4:**
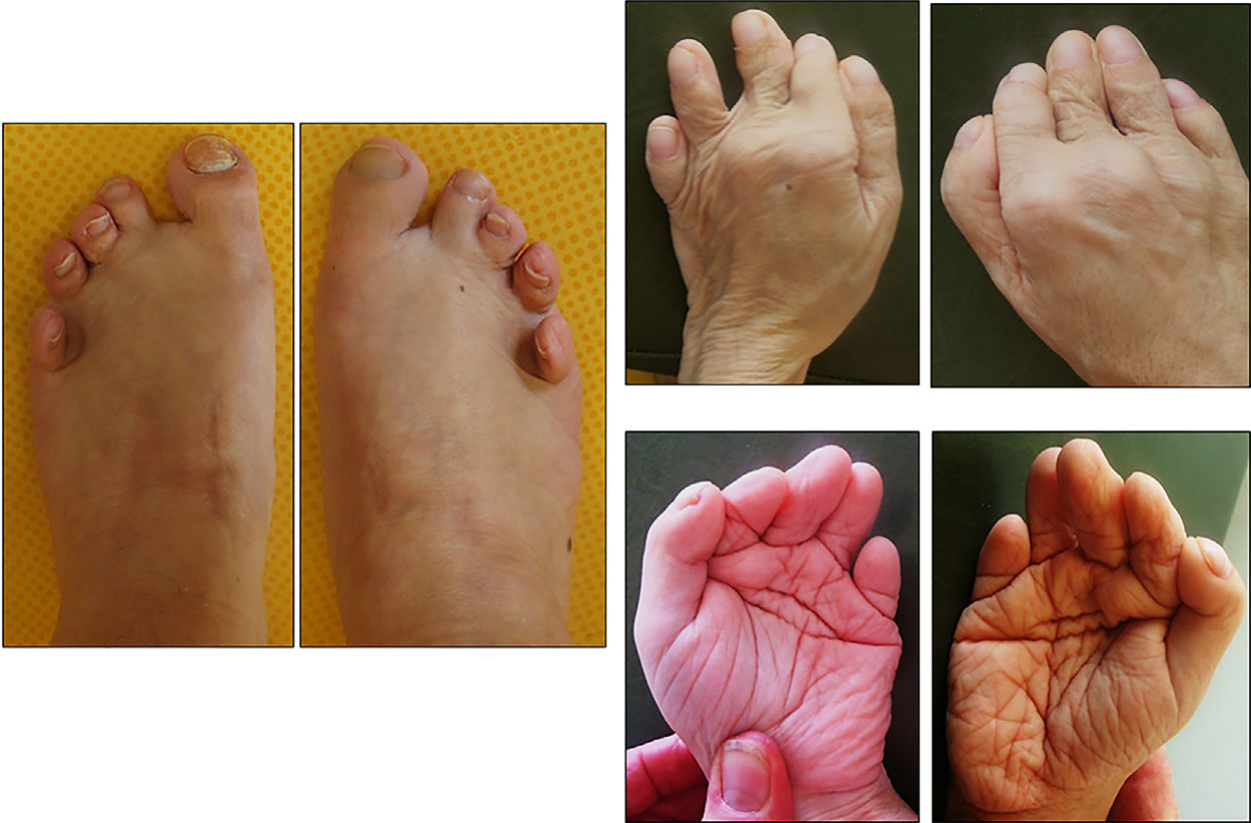
Patient 2: Hands and feet showed short toes and fingers, sandal gaps, deep palmar creases, and hands acrogeria with skin wrinkles. [Color figure can be viewed at http://wileyonlinelibrary.com]

**Figure 5:**
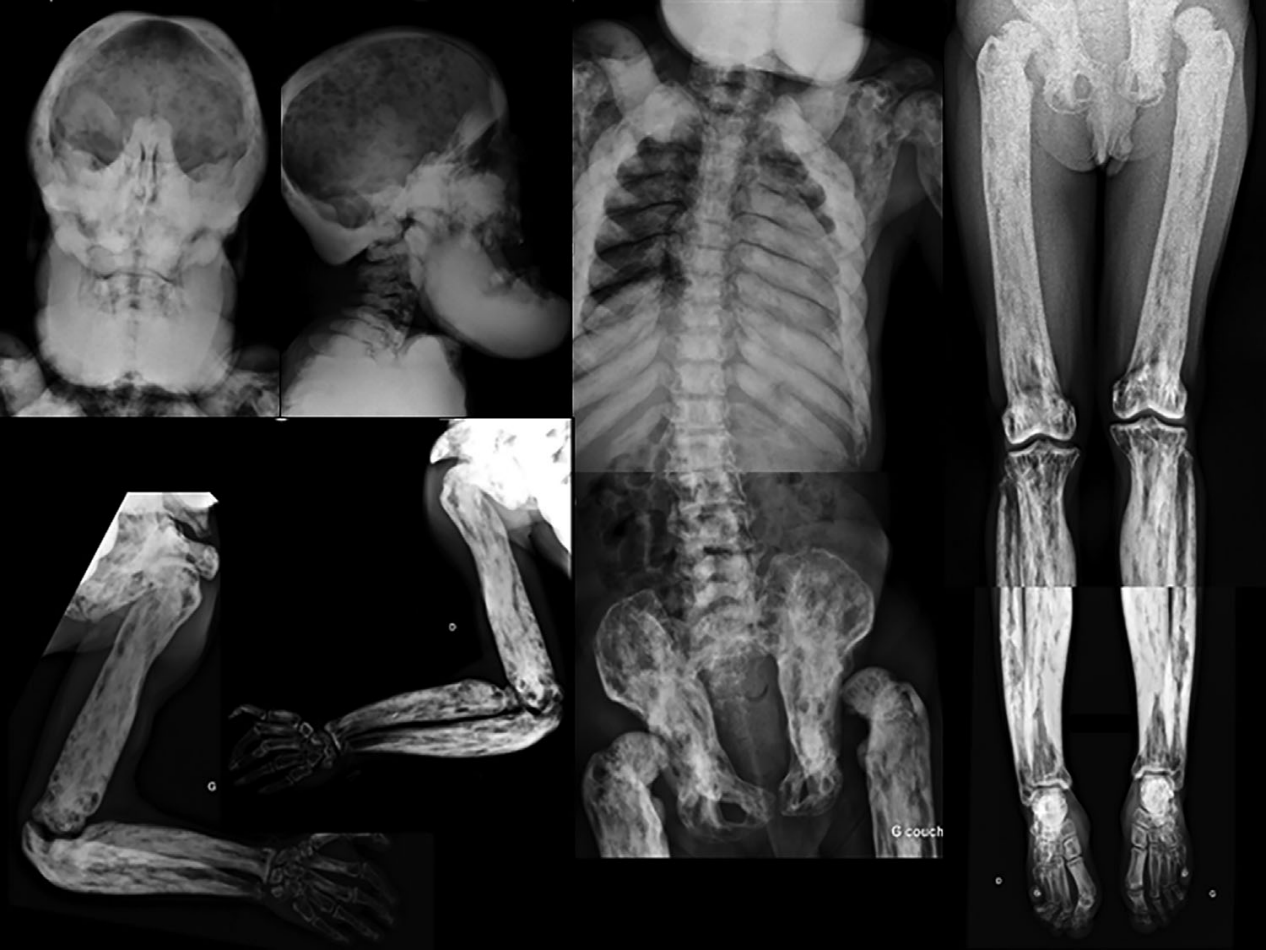
Skeletal survey of patient 2. The patient has generalized hyperostostic dysplasia, an enlarged mandible, shortened broad ribs, bilateral hip dislocation, cortical thickening of the long bones, very short metacarpals and symphalangism by fusion of proximal and intermediate phalanges of 2nd to 5th ray, similar to those observed in patient 3 (not shown)

Patient 2 was a male born at 41 weeks of gestation, with a birth weight of 2310g (−2.2 SD), a length of 46 cm (−3 SD), and OFC of 32 cm (−3 SD). Widespread thin skin wrinkles were present all over the body and the skin was translucent. Facial dysmorphism comprising broad forehead, prominent eyes, telecanthus, medial crease of the philtrum, thin vermilion of the lips, and retrognathia was observed (Figure 2). His developmental milestones were severely delayed: he walked without assistance at 40 months and learned only a few words. Generalized seizures without fever started at 4 years and were responsive to antiepileptic drugs. Fontanelle closure was delayed until 9 years. When examined in adulthood, a coarse long face, tall and broad chin with marked prognathism, broad forehead, highly arched thick eyebrows, telecanthus, high and broad nasal bridge, smooth philtrum and thin vermilion of the lips were observed. Prominent veins and limited extension of elbows were also noted (Figure 3) alongside with major brachydactyly of fingers and toes, a sandal gap, deep palmar creases, and hands acrogeria with skin wrinkles on their dorsal aspects (Figure 4). His progressive cranial bone deformity was associated with profound deafness. At 26 years of age, OFC was 54 cm (−2 SD); height was 123.4 cm (− 8.6 SD) an weight 33.8 kg (−5 SD). On skeletal survey a severe and generalized hyperostostic dysplasia was observed (Figure 5) with thickening of cranial vault, marked sclerosis of the skull base and facial bones, a disproportionately enlarged mandible, shortened broad ribs, hyperostotic clavicles and scapulae, dense vertebral bodies, hyperostotic iliac wings, and bilateral hip dislocation. Long bones exhibited diaphyseal undermodeling, marked hyperostosis, and cortical thickening. A severe shortening of the metacarpal bones especially those of the 4th and 5th fingers, a bilateral overdevelopment of the 1st ray proximal phalanx and the fusion of proximal and intermediate phalanges of 2nd to 5th ray was observed.

### 2.4 Patient 3

**Figure 6:**
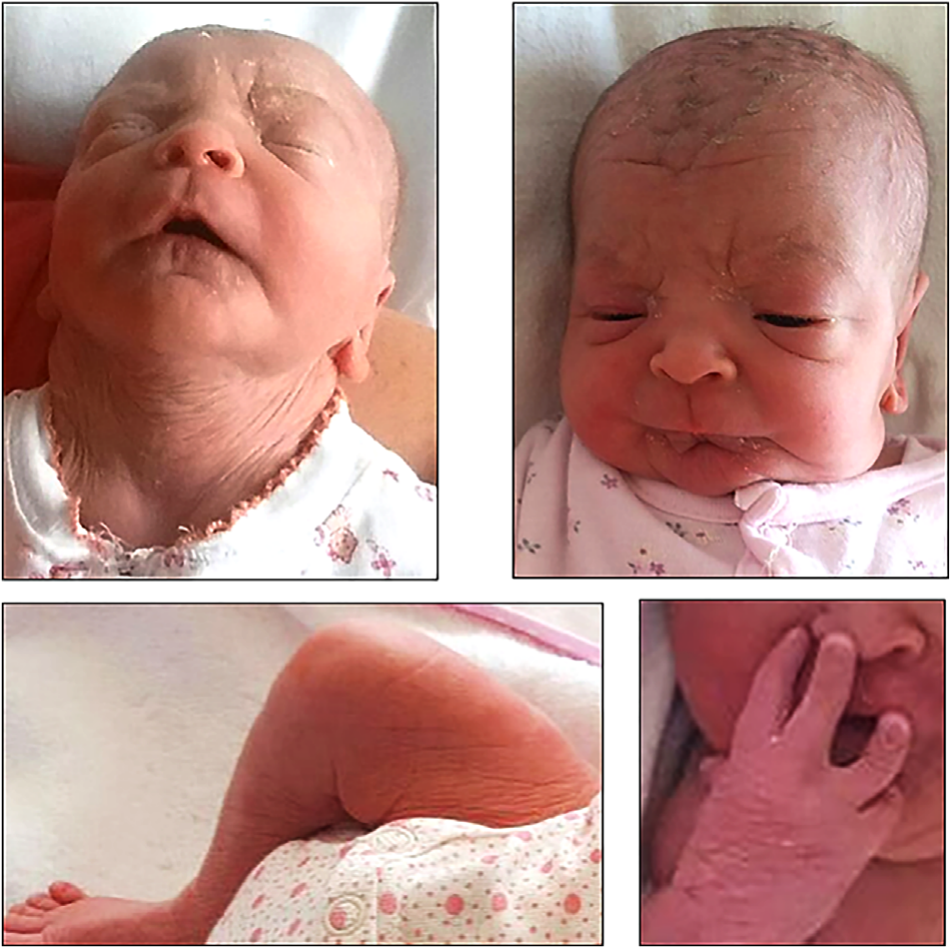
Patient 3 at birth. Prominent wrinkling of the skin in the neck, forehead, chin, and right thigh can be seen. The patient has a long philtrum with a medial crease, thin vermilion of the lips, telecanthus, sagging cheeks, and retrognathia which are part of the distinctive facial dysmorphism. Moderate brachydactyly of all fingers was noted, particularly affecting the 4th and 5th rays, also with interdigital webbing and clinodactyly. [Color figure can be viewed at http://wileyonlinelibrary.com]

Patient 3 was the second child of healthy non‐consanguineous Romanian parents aged 38 and 36 years. A nuchal edema prompted prenatal cytogenetic analysis indicating a 46, XX fetal karyotype. Delivery by caesarean section at 38 weeks of gestation gave birth to a female baby with normal weight (2570 g; −1.4 SD) and length (51 cm; +0.3 SD), and a small head size (31 cm; −2.5 SD). Skin wrinkles were present all over her body and head, giving her a progeroid appearance with sagging cheeks. A large anterior fontanelle and facial dysmorphism including telecanthus, a long philtrum with a medial crease, downturned corners of the mouth, thin vermilion of the lips, and retrognathia were observed (Figure 6). Moderate brachydactyly of all fingers was noted, affecting particularly the 4th and 5th rays, with interdigital webbing, and clinodactyly. A persistent foramen ovale was diagnosed on heart ultrasound. At 3 months, a deceleration of head growth was noted with an OFC at 34 cm (−5.9 SD) as well as short fingers and symphalangism of III, IV, and V digits. At the age of 17 months she showed a mild developmental delay. After exclusion of a congenital cutis laxa gene panel by NGS, LMS was considered based on the clinical presentation.

## 3 RESULTS

Sequencing of *PTDSS1* identified a heterozygous missense c.794T>C (p.Leu265Pro) mutation in exon 7 in patient 1, a heterozygous missense c.284G>T (p.Arg95Leu) mutation in exon 3 in patient 2, and a heterozygous missense mutation c.806C>T (p.Pro269Leu) in exon 7 in patient 3. None of the variants were present in public (gnomAD, ExAC, Exome Variant Server, dbSNP) or in‐house control databases. All three variants were predicted damaging or equivalent by SIFT, PolyPhen, MutationTaster, Provean, and other tools using the Variant Effect Predictor (http://www.ensembl.org/info/docs/tools/vep/index.html). De novo inheritance was confirmed in patient 3. In the two other cases, parental samples were not available.

## 4 DISCUSSION

In the present report of three patients with congenital CL, we provide evidence for a fourteenth etiology underlying this cutaneous phenotype (Table 1). Gain of function mutations in *PTDSS1* underlie LMS, which is an extremely rare syndrome with only 16 sporadic cases reported in the literature between 1969 and 2015 (Lenz & Majewski, 1974; Majewski, 2000; Sousa et al., 2014; Tamhankar et al., 2015; Whyte et al., 2015). Clinical features of LMS include craniotubular hyperostosis, loose skin, progeroid appearance, marked growth failure, and moderate to severe intellectual disability. Loose atrophic skin or CL has been described in all reported patients.

**Table 1:** Differential diagnosis of congenital cutis laxa

	*FBLN5*	*FBLN4/EFEMP2*	*ELN*	*ATP6V0A2*	*PYCR1*	*ALDH18A1*	*RIN2*	*LTBP4*	*ATP6V1E1* and *ATP6V1A*	*ATP7A*	*GORAB*	*SLC2A10*	*PTDSS1*
Enlarged fontanelles				+	+	+							+
Short stature				+	+	+					+		+
Intellectual disability				+	+	+			+				+
Cortical/cerebellar malformations				+	+				+ (*ATP6V1A*)				
Pulmonary emphysema	+	+	+		+			+					
Bladder/intestine diverticulae	+							+		+			
Early demise	+	+		+				+	+				+
Facial dysmorphism	+	+		+		+		+	+			+	+
Pulmonary artery stenosis	+	+	+					+					
Aneurysms		+	+		+							+	
Arterial tortuosity		+				+				+		+	
Cardiac abnormalities									+				
Cataracts					+	+							
Macrocephaly and sparse hair							+						
Microcephaly and epilepsy				+	+								
Hypotonia					+	+			+				
Skeletal features	Bone fragility			Scoliosis, hip dislocation	Hip dislocation	Hip dislocation	Scoliosis			Occipital exostoses	Osteoporosis and fractures		Craniotubular hyperostosis, brachydactyly, hip dislocation, kyphoscoliosis
Other					Choreoathetosis					Pili torti			

Sousa et al. (2014) identified causative heterozygous missense de novo mutations in *PTDSS1* in five unrelated LMS‐affected patients. The c.1058A>G; (p.Gln353Arg) mutation in exon 9 was identified in three previously described patients while two new cases harbored c.794T>C; (p.Leu265Pro) and c.805C>T; (p.Pro269Ser) mutations in exon 7. The p.Leu265Pro mutation corresponds to patient 1 in this report, while patient 3 in this report has c.806C>T; (p.Pro269Leu) resulting in a different amino acid substitution at the same Pro269 position to another LMS patient reported by Sousa et al. (2014). Since then, two further LMS patients have been described, with mutations in exon 7 (Tamhankar et al., 2015; Whyte et al., 2015). This means that LMS is a very rare disease with our description here of the novel c.284G>T; (p.Arg95Leu), and c.806C>T; (p.Pro269Leu) *PTDSS1* mutation representing only the eighth and ninth patients with confirmed molecular diagnoses reported so far. Many of the reported mutations are recurrent at specific amino acid positions and all cluster in non‐transmembrane domains, which are found on the luminal side of the endoplasmic reticulum/mitochondria‐associated membranes. They also locate at or nearby to amino acid residues in which mutations were previously demonstrated to be activating in a study by Ohsawa et al. (2004) who studied PSS1 activity following systematic alanine mutagenesis in Chinese Hamster ovary (CHO) cells. Enzymatic studies in LMS patient fibroblasts confirmed similar findings, demonstrating increased synthesis of PS associated with resistance to PS feedback inhibition (Sousa et al., 2014). The c.284G>T (p.Arg95Leu) mutation is located in exon 3 and also corresponds to a distinct cytosolic non‐transmembrane domain. Interestingly, this mutation affects the same arginine residue (Arg‐95) that is altered in a CHO cell mutant named “29” isolated from CHO‐K1 cells in which the biochemical activating effect was first demonstrated (Hasegawa, Kuge, Nishijima, & Akamatsu, 1989; Kuge, Hasegawa, Saito, & Nishijima, 1998; Ohsawa, Nishijima, & Kuge, 2004). These earlier observations therefore strongly suggest that this mutation is also likely to cause a gain‐of function, as has been described for previously reported mutations (Sousa et al., 2014).

The current report aims at confirming LMS as one of the differential diagnoses of CL in the neonatal period. The two distinctive features in the neonatal period appear to be brachydactyly and facial dysmorphism including telecanthus and thin vermilion of the lips, slowly evolving in a quite different dysmorphism through progressive deformation of the skull and mandible. By contrast to some other types of CL characterized by bone fragility, LMS displays generalized hyperostosis (see Table 1).

Most reported patients did not survive until adulthood. Majewski, (2000) described a prolonged survival in a 30‐ year‐old female. This patient had intellectual disability, marked growth failure (final height at 120 cm), hypertelorism, strabismus, large and prominent eyes, thick lips and tongue, enlarged mandible, sparse subcutaneous fat, prominent veins, atrophic and weak skin, massive thickening of long tubular bones, kyphoscoliosis, limited supination and extension of the elbow joints, brachydactyly and syndactyly.

In our patient 2, at 26 years of age, we notably observe enlargement of the mandible determining an elongated appearance of the face and a wide nasal root, which appears highly set. In contrast, telecanthus, strabismus, prominent eyes and veins were present from childhood on. An interesting point is the regressive course of CL over the years similar to what has been observed in most other subtypes of CL.

Although it might be difficult to draw general statements concerning handling and follow‐up of such a very rare condition with only nine *PTDSS1* mutation confirmed patients known until now, the follow‐up of such patients should comply with general recommendations concerning severely to profoundly disabled individuals. Nonetheless, special attention should be paid to their progressive skeletal dysplasia, and particularly to disproportionate growth of the mandible and the corresponding impairment of feeding, teeth malocclusion and pain. Consequently periodic consultations with the orthopedic surgeon and the dentist or the maxillofacial surgeon are recommended.

Skeletal deformities evolve similarly to those of a lysosomal storage disorder, progressing into dwarfism with facial coarsening over two decades. The marked prognathism displayed by our patient 2 resembles that observed in sclerosteosis (OMIM 269500), van Buchem disease (OMIM 239100), or craniodiaphyseal dysplasia (OMIM 122860), the most severe and lethal form of craniotubular dysplasia, which are all caused by *SOST* deficiency (Kim et al., 2011). Although no functional link between *SOST* and *PTDSS1* has yet been established, the phenotypic overlap suggests activation of Wnt signaling pathway, which normally promotes bone formation, and underlies high bone mass disorders (MacFarlane et al., 2017).

In conclusion, we report here three patients with LMS and heterozygous mutations in *PTDSS1*. We describe an adult phenotype and two novel *PTDSS1* mutations. We suggest that LMS should be considered in the differential diagnosis of a newborn with CL. Brachydactyly and facial dysmorphism should guide the correct diagnosis at this period. While the CL improves with age, in contrast to other CL‐associated syndromes, LMS evolves to a severe skeletal dysplasia with major growth failure alongside with severe intellectual disability. Therefore this syndrome can be easily distinguishable from other forms of CL at later stages.

## CONFLICTS OF INTEREST

The authors declare no conflict of interest.
